# Artificial neural network for enhancing signal-to-noise ratio and contrast in photothermal optical coherence tomography

**DOI:** 10.1038/s41598-024-60682-7

**Published:** 2024-05-04

**Authors:** Mohammadhossein Salimi, Nima Tabatabaei, Martin Villiger

**Affiliations:** 1 Department of Mechanical Engineering, Lassonde School of Engineering, York University, Toronto, ON M3J 1P3 Canada; 2 Center for Vision Research, York University, Toronto, ON M3J 1P3 Canada; 3 Harvard Medical School, Wellman Center for Photomedicine, Massachusetts General Hospital, Boston, MA 02114 USA

**Keywords:** Biophysics, Mathematics and computing, Optics and photonics

## Abstract

Optical coherence tomography (OCT) is a medical imaging method that generates micron-resolution 3D volumetric images of tissues in-vivo. Photothermal (PT)-OCT is a functional extension of OCT with the potential to provide depth-resolved molecular information complementary to the OCT structural images. PT-OCT typically requires long acquisition times to measure small fluctuations in the OCT phase signal. Here, we use machine learning with a neural network to infer the amplitude of the photothermal phase modulation from a short signal trace, trained in a supervised fashion with the ground truth signal obtained by conventional reconstruction of the PT-OCT signal from a longer acquisition trace. Results from phantom and tissue studies show that the developed network improves signal to noise ratio (SNR) and contrast, enabling PT-OCT imaging with short acquisition times and without any hardware modification to the PT-OCT system. The developed network removes one of the key barriers in translation of PT-OCT (i.e., long acquisition time) to the clinic.

## Introduction

Optical coherence tomography (OCT) is a biomedical imaging method based on interferometric detection of light backscattered by biological tissues^1,2^. OCT is routinely used both in a clinical and preclinical setting. In ophthalmology, OCT is the gold standard for diagnosis and monitoring of a broad spectrum of retinal diseases^3,4^. In interventional cardiology, intravascular OCT (IV-OCT) is used to guide percutaneous coronary interventions and serves as a powerful research tool to study the pathophysiology of coronary atherosclerosis^5,6^. The image contrast in OCT tomograms originate from elastic scattering of light by tissue microstructure and its refractive index inhomogeneities. As such, OCT images are inherently insensitive to the molecular make-up of the interrogated tissue. While many diseases eventually result in a structural alteration, additional compositional or molecular contrast would offer increased specificity and could enable earlier detection of diseases. For example, detection of lipid content in coronary atherosclerosis^7^ or calcium deposits in dental caries^8^ enable early detection of the diseases.

Photothermal (PT)-OCT is a functional extension of OCT that integrates narrowband PT laser(s) into the OCT system to enable pump-probe detection of absorption by a molecule of interest (MOI) within the tissue^9^. The selective absorption of the PT laser by the MOIs causes local tissue heating, that in turn results in a change of the optical path length (OPL) through a variation of the refractive index and physical expansion. The typical variation in OPL is on the order of tens of nanometers and can be sensed/detected through monitoring of OCT’s phase signal^9,10^. A key advantage of PT-OCT is its inherent ability to produce depth-resolved maps of light absorption that are automatically co-registered with OCT structural tomograms, offering refined insight into the spatial distribution (and potentially composition) of MOIs.

To date, promising PT-OCT results have been reported for the detection of a variety of MOIs, either through labeling with exogenous contrast agents^11,12^ or in a label-free fashion^13–15^, in-vivo^16,17^ and ex-vivo^18^. Despite these encouraging results and technological advancements, reliable deciphering of MOI-specific information from PT-OCT datasets remains challenging. The first and foremost fundamental challenge for PT-OCT is phase signal SNR which hinders the ability to reliably detect small phase signals within clinically acceptable measurement times. Other specific challenges include disentangling the observed photothermal signals from influencing system and sample parameters^10,13^ and accounting for the phase shadowing along the depth^19^.

Given that most OCT systems have Michelson configuration, unavoidable relative thermal and other perturbations in sample and reference arms cause phase fluctuations that result in a phase noise-floor substantially above the theoretical limitation imposed by the OCT SNR. Common-path OCT configurations achieve improved phase stability^20,21^, but at the cost of limiting the scanning range, the ability to control the reference beam power, and the working distance. Due to these limitations and the system complexity of common-path configuration, non-common-path OCT configurations with SNR limitations are still widely used for PT-OCT imaging.

Conventional PT-OCT modulates the intensity of the PT laser with a known carrier frequency. The received OCT phase signals are then demodulated at this frequency, which enables separation of the signal from noise^9,17,22^. For this approach to be effective, however, phase signals need to be acquired for long durations over many carrier modulation cycles. This requirement significantly slows down PT-OCT imaging and limits its translation into a clinical setting. PT-OCT variants utilizing optical demodulation of phase signals such as poli-OCM^22^ and poli-OCT^17^ were developed to increase the imaging speed, but to do so high carrier frequencies ought to be used which limited the effective imaging depth.

Recent advancements in the fields of artificial intelligence and machine learning have opened the door for computational enhancement of imaging system performance without the need for making modifications to system hardware. For example, for atomic force microscopy (AFM), Borodinov et al.^23^ improved the detection limit by more than an order of magnitude using a hybrid deep learning model. In OCT, deep learning (DL) models have been used for various purposes, such as segmentation^24–26^ and reconstruction of OCT structural tomograms^27,28^, dispersion compensation^29^, diagnosis^30^, and classification of retinal disease^31,32^, or automated noise and artifact removal^33,34^. Deep learning also has been used in functional extensions of OCT. In optical coherence elastography, Neidhardt et al.^35^ applied a DL model to quantify the mechanical elasticity of samples. Kim et al.^36^ reported an improvement in the imaging rate for OCT-Angiography using DL models.

In this paper, we employ a neural network to improve the SNR and contrast in PT-OCT images taken by a conventional, non-common-path OCT system. The neural network is trained to predict the amplitude of the photothermal phase modulation from a temporal signal trace much shorter than the one used to define ground truth (GT). The performance of the model is verified with controlled phantoms, lipid-containing biological tissues, and a human aorta sample. Although machine-learning models have previously been employed for classification purposes of PT-OCT results^37^, to the best of our knowledge, this is the first report on utilizing deep learning in the field of PT-OCT for enhancing SNR and image contrast which in return lowers the required acquisition time.

## Results and discussions

### Deep learning strategy for SNR/contrast improvement in PT-OCT images

**Figure 1:**
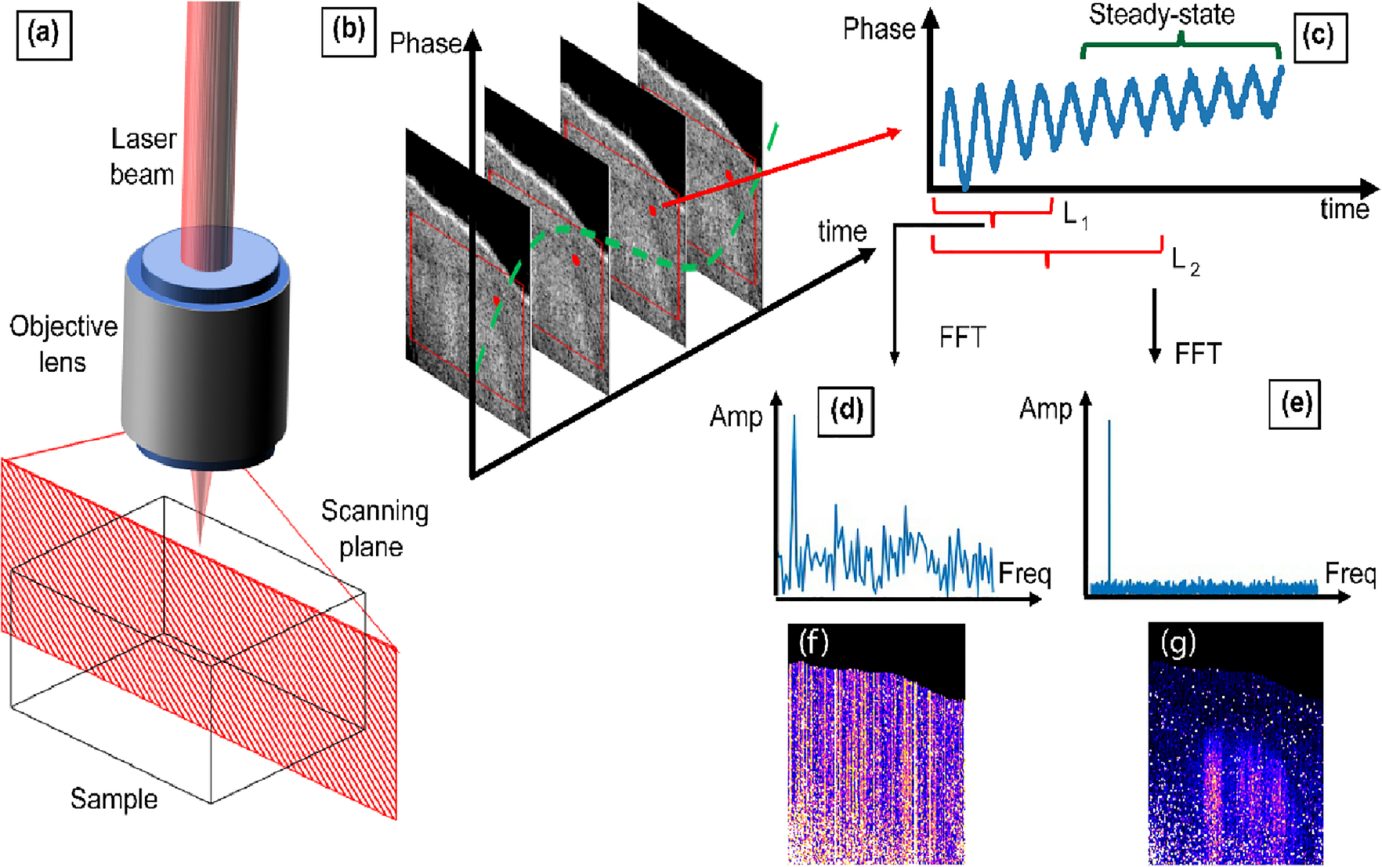
(**a**) Schematic of PT-OCT beam and cross-sectional imaging geometry. (**b**) Repeated imaging of the cross-section over time reveals harmonic modulation of the phase signal at a given sample point (red dot) due to illumination with a modulated of PT laser beam, plotted in (**c**). L1 and L2 represent a short and a longer segment of the acquired signal trace. FFT spectra of (**d**) the short L1 signal segment with low SNR and (**e**) the longer L2 signal segment with enhanced SNR; the amplitude of the harmonic peak at the PT laser modulation is used to generate PT-OCT images. PT-OCT images corresponding to (**f**) short L1 segments and (**g**) longer L2 segments, showing significant image enhancement when acquisition time is dramatically increased.

In PT-OCT, to detect a MOI in the sample, an intensity-modulated PT laser, with a wavelength set to the absorption band of the MOI, is added to a conventional OCT system. Figure 1a schematically shows the scanning plane that is generated by scanning the combined beams in one lateral direction over the sample surface. Absorption of the modulated PT light by the MOI results in generation of local modulated temperature field (aka. thermal wave field), leading to local modulation of the optical path length (OPL) near the MOI. Temporal OCT phase signals can screen the MOI-induced OPL changes; however, since such phase modulations are generally weak (especially for endogenous MOIs), PT-OCT utilizes phase-sensitive detection approaches such as lock-in demodulation for retrieving MOI absorption information from noisy signals^9^. That is, since the intensity of PT excitation is modulated at a known frequency, the acquired temporal phase signals are demodulated at the known frequency either via dual-phase demodulation or complex fast Fourier transform (FFT; Fig. 1b–e) to retrieve the amplitude of the PT-induced phase modulations. The performance of this detection approach is directly proportional to the length of the acquired photothermal responses, as longer acquisition times allow for better suppression of background noise through averaging. Figure 1c depicts an experimental phase signal of a phantom sample over several modulation cycles. L_1_ and L_2_ segments correspond to 3 cycles and 8 cycles of the signal. Signal spectra corresponding to L_1_ and L_2_ (Fig. 1d, e), show clear enhancement of SNR for the longer signal which ultimately leads to generation of a PT-OCT image with better SNR and contrast (Fig. 1g versus f). Such enhancement in image contrast ultimately translates to the ability of detecting fine spatially resolved details of the MOI, albeit at the cost of longer acquisition time (i.e., lower imaging speed).

The plots in Fig. 1 illustrate the compromise between image quality and speed which is inherent to phase-resolved measurement techniques. In this study, the idea is to design a DL model that takes noisy PT-OCT signals of limited acquisition length as input (e.g., Fig. 1d) and predicts PT-OCT images with enhanced SNR and contrast that normally can only be achieved through processing of long-acquisition signals (e.g., Fig. 1g). In other words, the network is fed with the short signal traces (L_1_ in Fig. 1c) and is trained to predict the pixel amplitude of the high SNR image (Fig. 1g). The underlying assumption of the current work is that training with experimental datasets enables the network to learn additional features of the PT-OCT signal such as the transient response of the signal (i.e., rate of gradual bulk heating ignored in lock-in demodulation approach^38^) to compensate for the shorter available signal trace. This may be specifically helpful in setups with inferior phase stability, such as Michelson-based interferometers, that are inherently prone to phase noise.

### Network performance on phantoms

**Figure 2:**
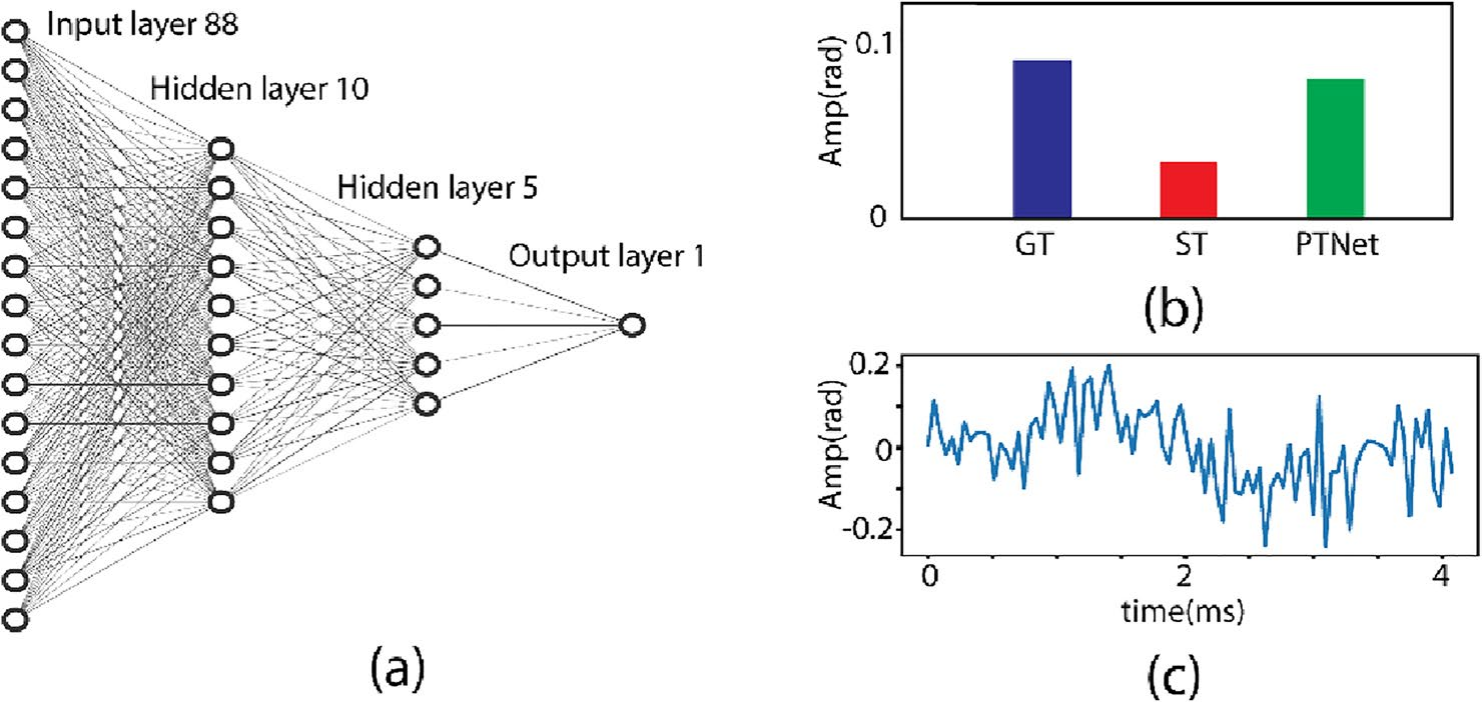
(**a**) The structure of the neural network. (**b**) Representative network inference result. Blue ground truth (GT) bar is calculated using conventional PT-OCT signal processing of the full/long signal trace; Red bar represents calculated amplitude from a two-cycle-short signal trace with conventional PT-OCT signal processing; Green bar shows signal amplitude predicted by the trained network (PTNet) when fed with a two-cycle-short signal trace. (**c**) Phase signal of the underlying short signal sequence (88 points).

Theoretical models of PT-OCT show that the power of the PT laser on the sample, the modulation frequency of PT laser amplitude, and the location of sample with respect to the OCT system focal plane are among significant parameters affecting the acquired PT-OCT signals^10,19^. In light of such theoretical works, a polydimethylsiloxane (PDMS) based phantom was designed for creating the training datasets. The PDMS sample was subsequently imaged under various powers of the PT laser, at various sample distances to the system focal plane, and at two different modulation frequencies of the PT laser (500 Hz and 4000 Hz). The captured data at each modulation frequency were used to train the corresponding deep network. The two networks had the same fully connected configuration (Fig. 2a). This network structure stemmed out of iterative optimization of the network architecture in terms of denoising performance. The input to the optimized network is a short trace of OCT phase signal containing the first 88 datapoints. The output is the prediction of the PT-OCT amplitude, as if conventional phase sensitive detection was carried out on an OCT phase signal containing 864 datapoints (i.e., GT). Note that at a modulation frequency of 500 Hz and at OCT A-line rate of 21,600 Hz, the signals with 88 and 864 data points are almost equal to 2 and 20 modulation cycles, respectively, corresponding to an order of magnitude difference in the acquisition time.

To demonstrate the performance of the trained network, the results of network’s inference and conventional PT-OCT signal processing for a sample OCT phase signal are depicted in Fig. 2b. The original signal has a length of 864 datapoints, leading to PT-OCT signal amplitude of 0.093 when processed with conventional PT-OCT algorithms (aka. GT PT-OCT amplitude value; blue bar in Fig. 2b). When a short trace (ST) of the original signal cut from the first 88 datapoints (Fig. 2c) is processed with the conventional algorithm, a significantly lower PT-OCT amplitude of 0.031 is obtained as highlighted by the red bar in Fig. 2b. However, when the same ST signal is fed to the network, a PT-OCT amplitude of 0.088, comparable to the GT reconstruction is obtained, green bar in Fig. 2b. This suggests that the trained neural network can effectively predict the long-signal-trace GT signal amplitude from the short signal trace.

**Figure 3:**
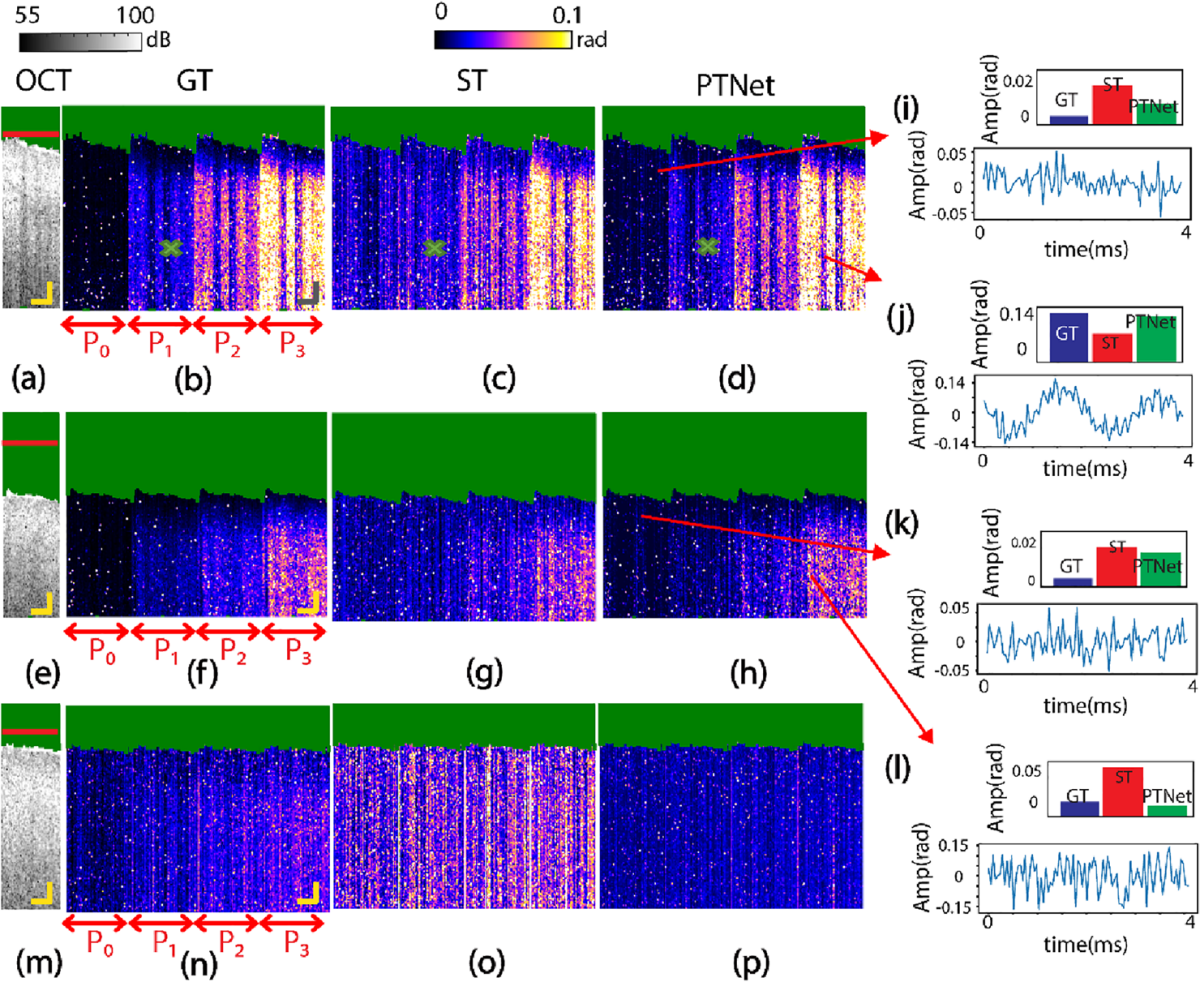
PT-OCT images reconstructed with the neural network and conventional method, acquired in different experimental conditions. The PDMS sample in in-focus condition: (**a**) OCT image, (**b**) GT, (**c**) ST, and (**d**) PTNet. The PDMS sample in out-of-focus condition: (**e**) OCT image, (**f**) GT, (**g**) ST, and (**h**) PTNet; (**i**) to (**l**) representative visualizations of signal trace and GT, ST, and PTNet amplitudes for single pixels. The PDMS sample measured at 4000 kHz: (**m**) OCT image, (**n**) GT, (o) ST, and (**p**) PTNet images. The green area in these panels represents intensity-masked pixels; Red lines in OCT structural images denote the system’s focal plane. Scale bars = 100 μm.

To show the performance of the networks with respect to in and out of focus imaging scenarios, a series of experiments were carried out on PDMS samples at 500 Hz. The results of a hold-out dataset captured from the PDMS phantom are plotted in Fig. 3a–l. Here the sample is imaged at different PT power levels of 0, 1, 2.25, and 3.5 mW (marked with P_0_ to P_3_ in Fig. 3b, respectively, to simulate different concentrations of absorbers). Qualitative assessment of the results suggests that the reconstructed image (i.e., PTNet; Fig. 3d) is more similar to the GT (Fig. 3a) than the PT-OCT image obtained from conventional PT-OCT signal processing of the ST signal (Fig. 3c). For instance, although the warp texture (marked by the green cross in Fig. 3d) is hidden in the ST image of Fig. 3c, it can be clearly seen in the PTNet and GT images. Moreover, the noise floor (P_0_ regions) in the PTNet image is significantly lower than that of the image obtained by the ST signal. This low noise floor improves the contrast in the network images, particularly between the P_0_ and P_1_ regions. To verify the performance of the network in “out of focus” condition, the PDMS sample was imaged approximately 200 μm out of focus. The OCT and PT-OCT results are depicted in Fig. 3e–h. The red lines in OCT structural images represent the position of the focal plane. Similar to the in-focus condition, a greater similarity can be seen between PTNet image (Fig. 3h) and the GT (Fig. 3f), particularly in P_0_ and P_1_ regions. Panels (i) to (l) include visualization of signal traces along with calculated and predicted PT amplitudes for single pixels. Panels (i) and (l) represent examples of good predictions, while panels (j) and (k) show poor predictions of the network.

To investigate the effect of PT laser modulation frequency (i.e., need in conventional PT-OCT for increasing imaging speed) on the performance of the network, an additional experiment was carried out at a higher modulation frequency of 4000 Hz. The network was re-trained using training data at this higher modulation frequency, following the same methodology as for modulation at 500 Hz. The OCT and PT-OCT results are depicted in Fig. 3m–p. As seen, the PT-OCT signal amplitudes in the GT (Fig. 3n) drop dramatically compared to those obtained at 500 Hz (Fig. 3b) due to characteristic frequency response of the thermal Green’s function and pink noise of the system^10^. Due to this dramatic reduction in SNR, the network shows poor performance in extracting the small signals from the time sequences and reconstructing the PT-OCT image (Fig. 3p). Nevertheless, unlike conventional PT-OCT signal processing (Fig. 3o), the network is quite successful in suppressing the baseline noise floor, which is not linked to absorption of PT light (Fig. 3p). This experiment highlights the major dilemma in PT-OCT imaging on the compromise between the SNR and imaging speed/rate. That is, at lower modulation frequencies, owing to the thermal Green’s function response, the amplitude of the PT-OCT signal is larger; however, phase-sensitive detection at lower frequencies is more prone to pink noise and requires acquisition of multiple cycles of the low frequency modulation, resulting in long acquisition times^10^. While the neural network can be trained to denoise the PT-OCT signal from a short, few-modulation-cycle signal sequence, it apparently fails to detect the weaker PT signal.

To quantify the performance of the network for the in-focus and the out-of-focus studies at 500 Hz, the Michelson contrast, MSE, and structural similarity (SSIM) metrics were calculated. The Michelson contrast values listed in Table 1 show that the network improves the contrast between regions in images, specifically between P_0_ (noise floor) region and other regions, although it does not quite achieve the same contrast as in the GT images. Note that since the P_0_ region can be seen as a non-absorber region of the PT light, the network offers significant improvement in contrast between absorber and non-absorber regions which is specifically helpful for determining the borders of absorber regions with higher accuracy.

**Table 1:** Michelson contrast of PT-OCT images (N = 20′000 signal traces).

Conditions		Contrast				
		P_0 _− P_1_	P_0 _− P_2_	P_0 _− P_3_	P_1 _− P_2_	P_2 _− P_3_
On focus	GT	61.8	77.4	86.7	29.9	28.2
	ST	6.9	23.5	47.4	16.9	26.8
	PTNet	35.4	57.7	75.6	28.0	31.7
Off focus	GT	42.9	62.8	77.8	27.1	29.4
	ST	0.4	15.9	32.17	16.3	17.1
	PTNet	12.2	39.4	61.3	28.2	29.3

Table 2 lists the MSE and SSIM values between the GT and the network output and the conventional reconstruction of the ST signals, respectively. The MSE between GT and the network images are approximately 10 times smaller than those between GT and ST conventional images. Similarly, the SSIM values between the GT and the network output, on average, are approximately 10% greater than the SSIM values between GT and ST conventional images. All quantitative performance metrics underline the network’s ability to reconstruct PT-OCT images with good similarity to the GT.

**Table 2:** MSE and structural similarity of PT-OCT images (N = 20′000 signal traces).

Conditions		MSE					SSIM				
		P_0_	P_1_	P_2_	P_3_	Overall	P_0_	P_1_	P_2_	P_3_	Overall
On focus	GT-ST	0.0025	0.0105	0.0139	0.0231	0.0167	51.2	82.2	82.9	84.6	77.0
	GT-Net	0.0014	0.0024	0.0014	0.0016	0.0017	83.4	88.5	91.3	92.3	89.2
Off focus	GT-ST	0.0132	0.0091	0.0111	0.0113	0.0107	63.0	83.3	84.3	85.1	80.4
	GT-Net	0.0018	0.0019	0.0005	0.0011	0.0013	88.1	91.3	92.5	89.8	90.8

It is worth pointing out that both the ST signal and the PTNet are closer to the GT, and to each other, with increasing PT laser power. The possible reason behind this trend is that by increasing the PT power, the SNR of the PT-OCT signals increases, therefore, the influence of noise on the signal will be less. However, the SNR of PT-OCT signals obtainable from endogenous tissue constituents is normally low (e.g., collagen in cartilage^15^ or melanin in retina^39^). To increase the SNR in such cases, either exogenous labels such as gold nanoparticles can be used; or the power of PT laser should be dramatically increased. Both approaches, however, are not applicable to many in-vivo scenarios because of potential for tissue damage or complexities and toxicity considerations of administering exogenous labels. As such, predicting reliable PT-OCT signal amplitudes from poor SNR data is an inherent need for PT-OCT imaging which can be addressed to a great extent by the developed network based on the performance metrics of Tables 1 and 2. These results show that the network is specifically powerful in removing/denoising signals not attributed to absorption of PT light which ultimately translates to more accuracy of PT-OCT images by reducing the false positive readings.

### Network performance on tissue

**Figure 4:**
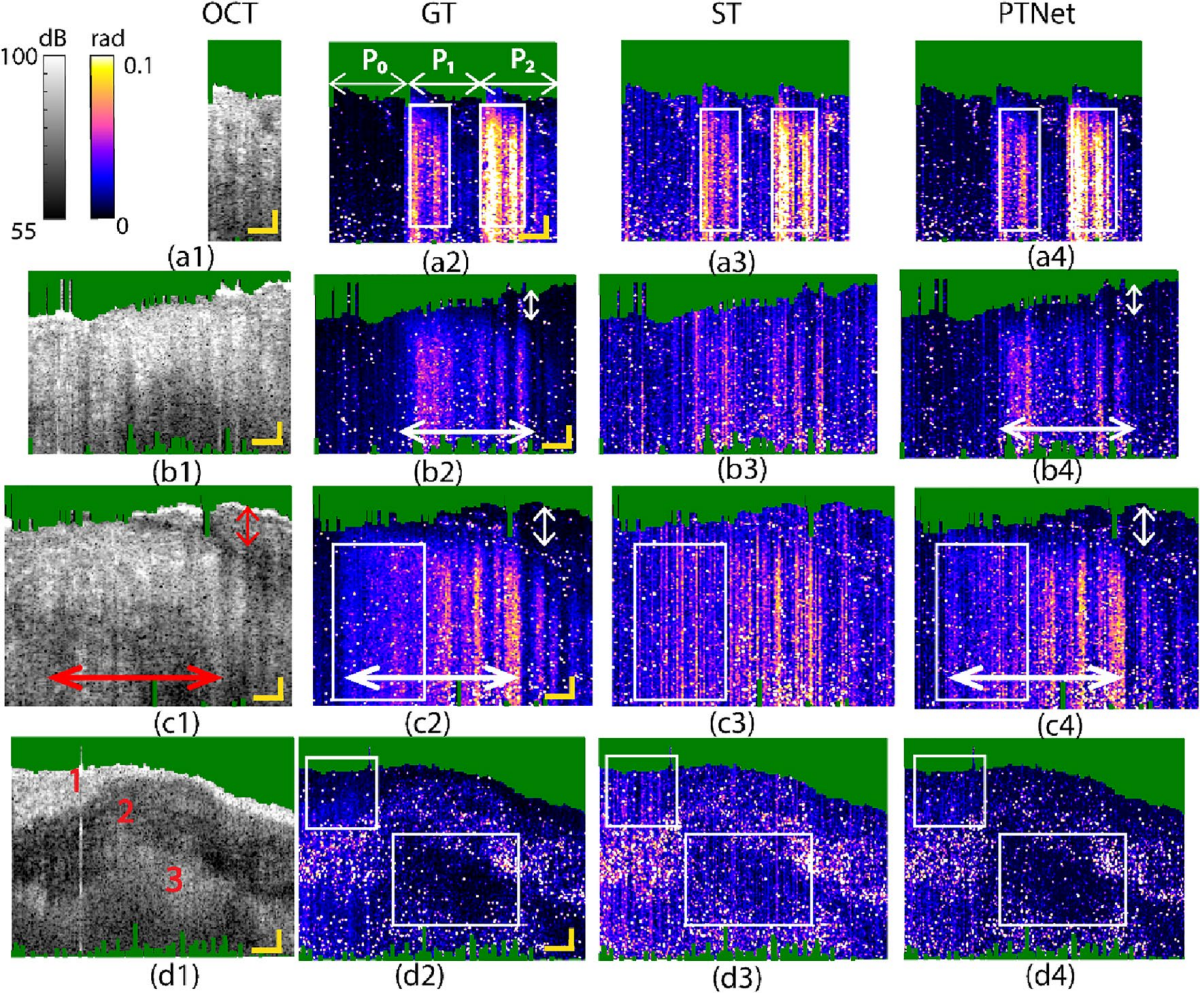
Captured OCT image; GT, ST, and PTNet images of (**a1**) to (**a4**) the adipose swine tissue (bacon) sample, (**b1**) to (**b4**) the artificial artery plaque sample, (**c1**) to (**c4**) another location of the artificial artery plaque sample, and (**d1**) to (**d4**) the human aorta sample. Panel a1 to a4; P0 to P2 correspond to PT power levels of 0, 1.6, and 3.2 mW. The green area in these panels represents intensity-masked pixels; Scale bars = 150μm.

To evaluate the ability of the previously trained network to generalize to biological samples, three different tissue samples were used for testing, including: an adipose swine tissue (Bacon), an artificial lipid-rich plaque, and a fresh human aorta sample with calcification. The network was previously trained with training data from the PDMS samples. Figure 4 displays inference with this network on these tissues. The samples were positioned to have the top surface in focus. In these experiments, the first 864 datapoints were selected in the 1000 datapoints of the GT signals, and the first 88 datapoints (corresponding to two modulation cycles at 500 Hz) were used as ST signals.

The adipose swine tissue was imaged at 3 different PT power levels. Comparison of PT-OCT images produced by the network and conventional PT-OCT signal processing (i.e., “ST”) with the GT images shows that the network is specifically successful in suppressing contributions of noise (P_0_ region of panel a4 vs. a3). Moreover, the absorber areas depicted by the white rectangles in inferred images show higher contrast between the area within the white rectangle and the background than those in the ST image, demonstrating the ability of the network to enhance contrast, which translates to easier and more reliable detection of the absorber region.

Similar observations can be made for the artificial lipid-rich plaque sample which contained a lipid pool beneath a tissue cap. Panels b1 to b4 and c1 to c4 depict results obtained from two different regions of the sample at the same PT laser power. Comparison of the PTNet images (b4 and c4) and ST images (b3 and c3) with the GT images (b2 and c2) again shows enhanced contrast, facilitating the detection of the border between the lipid region and the cap (white arrows). Determination of the extent and location of lipid underneath a fibrous cap is critical for assessing the risk of an atherosclerotic lesion to cause a future coronary event ^40–42^. Moreover, the texture details of the inferred images appear to be more consistent with those of the GT than the ST images. For instance, the selected white window in the ST image (c3) shows a region with relatively large amplitude of PT-OCT signals while such region in the PTNet image (c4) shows more resemblance to that in the GT image (c2).

The results of the human aorta tissue are shown in panels d1 to d4. The OCT image (d1) shows three distinct sample regions: (1) A scattering superficial layer, (2) an echolucent layer within the calcified area, and (3) a deeper slightly more scattering layer. The GT image of panel d2 suggests weak PT signals in the selected white windows of regions 1 and 3; similar weak signal levels are observed in the PTNet image of panel d4. In the ST image of panel d3, however, the selected windows contain erroneously large PT-OCT signals (mostly blue and purple colored pixels).

Although the network was trained with data from a PDMS sample containing absorbing dye, the results of Fig. 4 suggest that the network offers good performance in reconstructing PT-OCT images of biological tissues. This phantom-based study allowed us to generate a spectrum of PT-OCT signal amplitudes—from weak to strong—by adjusting phantom properties and experimental parameters. By ensuring that the received signal amplitudes fell within the range observed in biological tissues, our phantom-based approach closely mimicked real-world clinical scenarios. Although the PDMS phantoms and biological tissues might exhibit basic differences regarding their properties, the received PT-OCT signals share some non-trivial features. Apparently, the network learns PT-OCT signal patterns and characteristics that are independent of the sample properties and only related to the intrinsic OCT phase signal.

### Generalization experiments and input size dependence

The above experiments focused on analysis of the first two cycles of the acquired data, when the thermal field in the sample consists of a transient and a steady-state response. The transient response will disappear after a few modulation cycles, leaving only the steady-state response (Fig. 1c). The steady-state response consists of a modulating term (AC part) and bulk heating (DC part). The conventional lock-in method measures the AC part of the steady-state response and rejects its DC part. The first few modulation cycles are often ignored in conventional PT-OCT signal processing to eliminate the effect of the transient response^43,44^. However, recently we demonstrated that this transient response offers an alternative strategy for PT-OCT imaging as the transient response is also correlated with absorption of PT light by MOI^38^.

**Figure 5:**
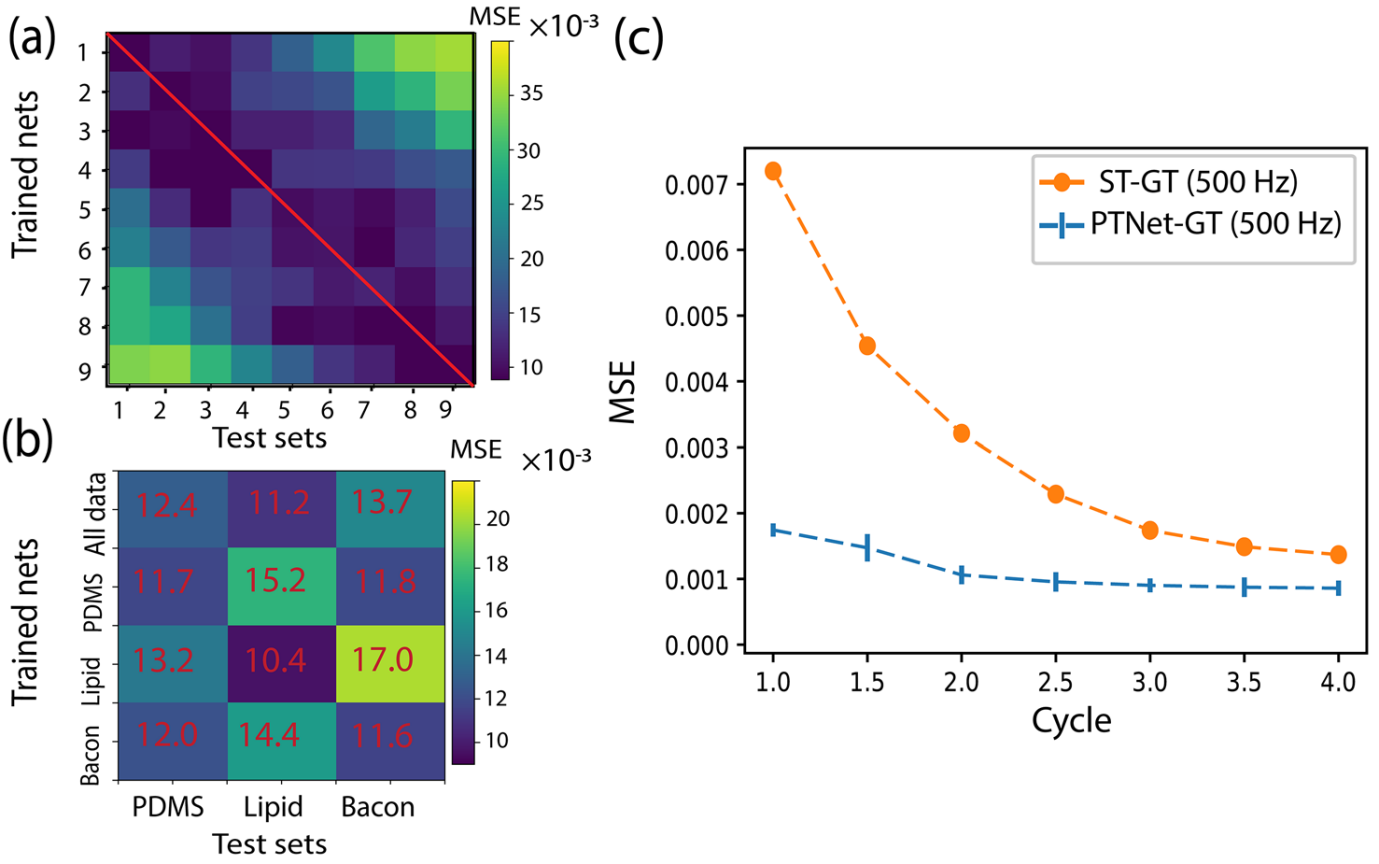
Generalization experiments and input size dependence. (**a**) MSE values of 9 networks trained with two cycle sequences occurring at different timepoints within the full response and tested on sequences from all timepoints. Datasets are from the PDMS sample. (**b**) MSE values of networks trained with data from various samples and tested on datasets from all samples. (**c**) MSE compared to GT in terms of length of input sequence for both conventional reconstruction (ST) and neural network reconstruction (PTNet).

To assess the sensitivity of the network to the transient thermal response, a study was designed using measurements of the PDMS phantom. To generate training and test datasets at different delays within the transient response, we split the full signal of 18 cycles into 9 consecutive groups of two cycles each. For example, group 2 contained the 3rd and the 4th cycles of modulation. Subsequently, 9 networks with the same structure were independently trained with these 9 training datasets, and each network was tested with unseen datasets from all groups. Note that the GT for all networks was identical and corresponded to conventional lock-in analysis of the entire 18-cycle signal. The MSE values between the network results and the GT are plotted in Fig. 5a, revealing a minimum error along the diagonal. This suggest that each trained network has a better performance when inferring from data originating from a similar time point within the PT modulation. For example, a network that is trained with the 3rd and the 4th modulation cycles has a better performance in predicting signals taken from the 3rd and 4th cycles. While we expected a clear time-dependence for the first few cycles, where the transient response dominates, it was unanticipated to find a similar dependence for the later cycles.

To further survey the generalization capability of the network to different samples, we independently trained three networks with the same structure using the datasets collected from the PDMS, the artificial lipid plaque, and the adipose swine (bacon) samples. In addition, a fourth network with the same structure was trained with a data pool containing all three sample types. The four trained networks were then tested on unseen data from all three samples. The MSE values between the GT and the network are plotted in Fig. 5b. The deviation of the MSE values between the different training/testing data sets is modest. Yet, training and testing on a single sample type offers best performance, in particular for the lipid sample. Training with the pooled data equalized performance across all sample types and suggest a limited generalization ability. Training on data from a specific sample type offers best performance. Owing to the experimental implementation of PT-OCT, it is relatively simple to generate GT data, and the simple network architecture facilitates re-training the network to optimize performance.

Lastly, we investigated the dependence of the network performance on the input signal length. We trained nets with different number of modulation cycles of the input signal (from 1 to 4). At an A-line rate of 21.6 kHz and modulation frequency of 500 Hz for the PT laser, each cycle consists of 44 datapoints (44/21,600≃2 ms). To accommodate the larger size of the input vector, we increased the network configuration to three dense hidden layers consisting of 40, 20, 5 neurons, that connect the input vector to a single neuron as the output. The GT for all networks were the same, taken from the first 20 modulation cycles of the captured experimental signals. The MSE between the network prediction and GT, and the ST signals and GT are plotted in Fig. 5c. With an increased number of modulation cycles used for training and conventional reconstruction, the MSEs compared to GT decreased. However, the MSE between the network and GT decreases by more than 50% when training with 2 cycles, compared to the use of only 1 or 1.5 cycles. Beyond 2 cycles, there is no significant improvement in the network performance. On the other hand, the MSE of the conventional reconstruction of the short sequence signal monotonically decreases. Extrapolating the curve for the conventional reconstruction beyond 4 cycles, the network’s MSE inferring from 2 cycles is matched with conventional reconstruction of 7.2-cycle signal segments. In other words, the MSE of a 2-cycle trained network is equal to conventional reconstruction of a signal with 7.2 cycles, when compared to GT.

The overarching goal of signal processing in PT-OCT is to demodulate a temporally fluctuating phase signal which is correlated with MOI concentration. Achieving this goal, however, is a very involved task because our recent theoretical models (and experimental results) show that PT-OCT signals are correlated not only with the concentration of MOI, but also with a broad range of system (e.g., pixel distance to OCT focal plane) and sample (e.g., light scattering) influence parameters^10,13^. Above studies suggest that machine learning-based strategies have the potential to overcome the SNR/contrast limitation of PT-OCT to a great extent and enable acquisition of PT-OCT images in time spans that are clinically acceptable. While we explored here the use of machine learning to achieve this goal, novel approaches using handcrafted algorithms may achieve similar speedups. A potential future extension of this work is development of similar DL models that are also informed by physics/theoretical models to not only enhance the SNR and contrast, but also consider the multifactorial effects of system and sample parameters when demodulating the temporal PT-OCT phase signals to yield further enhancements in PT-OCT images.

## Conclusions

This manuscript presented a strategy to improve SNR and contrast in PT-OCT images using a neural network. Conventional PT-OCT requires acquisition of a long signal trace to obtain high SNR and combat phase fluctuations typical in non-common path setups. By training a neural network to predict the conventional PT-OCT signal from only a short signal trace, we demonstrated the possibility of significantly improving the imaging speed in PT-OCT. In this strategy, relatively good generalization of the network in different sample and acquisition conditions comes with the simplicity of generating tailored GT data and retraining the network; therefore, it likely offers the most promising strategy to balance PT-OCT imaging performance with imaging speed, which remains one of the primary obstacles for practical applications of PT-OCT.

## Methods

### Setup and instrumentation

**Figure 6:**
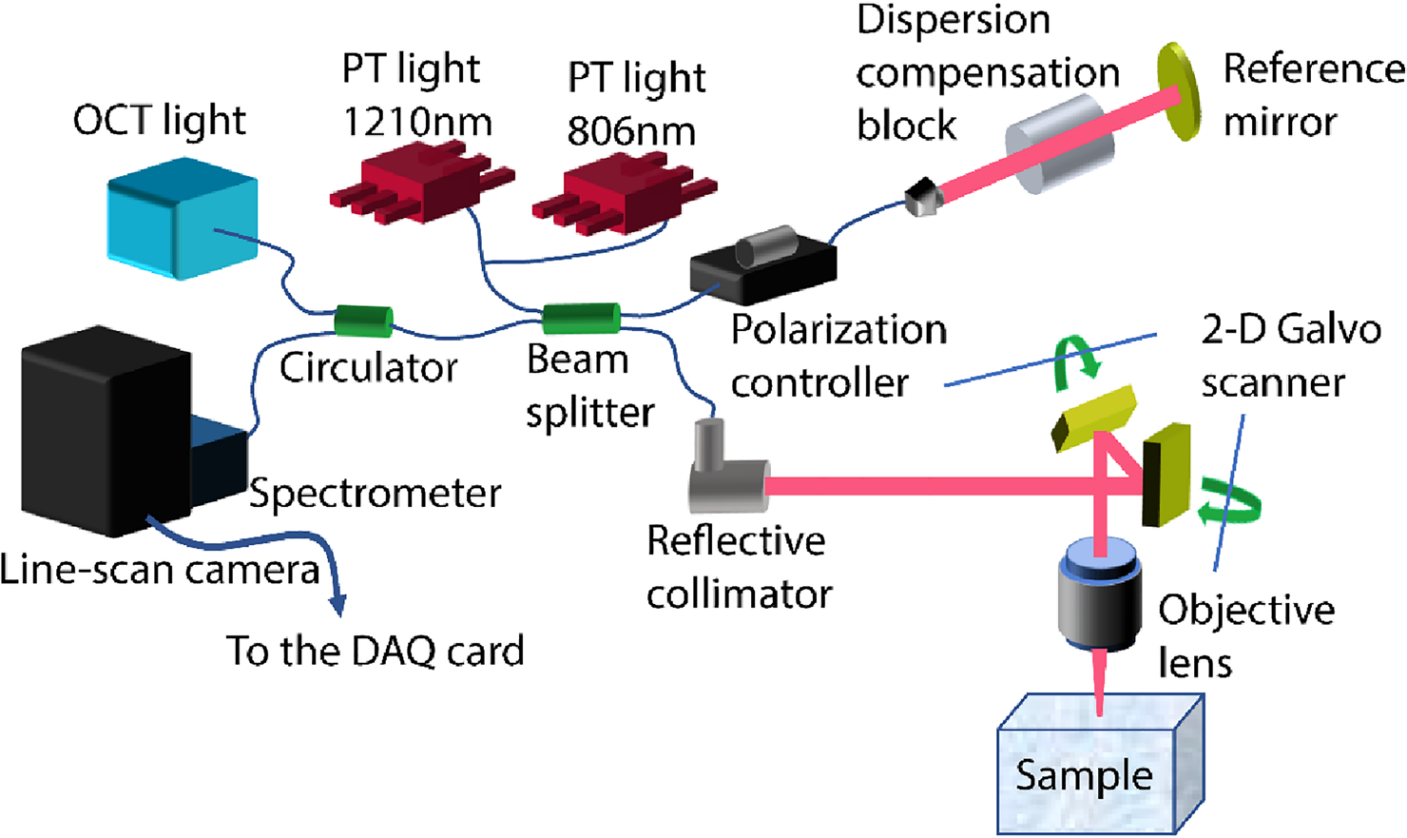
Schematic of the PT-OCT setup. The OCT system used an infrared superluminescent diode (Exalos; Switzerland) with λ0 = 1315 nm central wavelength, 115 nm full width at half maximum, and a maximal output power of 30 mW. Two narrow band laser diodes were used for photothermal modulation, emitting at 806 nm (max power = 30 mW; Thorlabs, U.S.) and 1210 nm (max power = 500 mW; Innolume, Germany), respectively.

The schematic and details of our setup is depicted in Fig. 6. The OCT light after passing through the optical circulator, is combined with the PT light in the 50/50 beam splitter. In this setup, two PT laser illuminating at 806 nm and 1210 nm are used. These two wavelengths are in the absorption peaks of lipid (1210 nm) and an exogenous dye (810 nm) targeted to the two MOIs in this study. Only one of the diodes was coupled into the OCT system at a time. The combined beams are divided into two beams via the beam splitter. In the sample arm, the light collimated light is focused on the sample surface after passing through the objective lens. Using the 2 degree of freedom Galvo scanner, the surface of the sample can be raster scanned. In the reference arm, the light is projected to the reference mirror. To prevent the dispersion effect, the dispersion compensation block is installed in the path of the light. The reflected-back light from the sample and the reference mirror, then is combined by the beam splitter and is delivered to the spectrometer by the optical circulator. The line scan camera digitizes the spectrum of the received signal and sends signals to the PC via the DAQ card.

The axial and lateral resolution of our OCT setup in tissue were measured as 9.88 μm and 11.35 μm, respectively. The system's phase stability, measured from the reflection of a glass slide was approximately 1.1 µm (14.6 millirad). The sensitivity was measured as 105 dB in air with a fall-off of 23 dB over 4 mm. The A-line rate of the system was variable, ranging from 9.6 to 147.8 kHz (for this study, it was set at 21.6 kHz). Additionally, the focal length of the objective lens is specified as 18 mm.

### Sample preparation

Phantoms were prepared with PDMS as the matrix. The two parts of PDMS (curing agent and the polymer) were mixed at a ratio of 1:10 by weight. Before creating the mixture, 25 mg of titanium oxide (Sigma Aldrich, U.S.) was added to 1 ml of the curing agent to induce scattering for OCT. Then, 25 mg of an absorber 806 nm dye (IR dye 806 nm, Sigma Aldrich, the U.S.) was dissolved in 1 ml of methanol. This solution was added to the suspension of scattering powder and curing agent. The suspension was stirred well for 20 min, before adding it to 10 g of the polymer in a petri dish (1 ml of curing agent + 10 g PDMS polymer), followed by additional stirring to make a homogenous phase of PDMS. After degasification in vacuum chamber, this mixture was poured into a mold and heated on a hot plate at 80 °C for 5 h. The sample was ejected from the mold after curing and cut into 3 × 3 cm^2^ pieces.

For the studies on biological tissues, 3 different samples were prepared, including: swine adipose tissue, an artificial atherosclerotic plaque made of bovine myocardium with lipid injected below the surface, and a fresh human aorta specimen. For the adipose tissue, a slice of fresh bacon (Kirkland) was cut into a 2 × 2cm^2^ piece with a surgical blade, selecting a region rich in fat (white layers). To make the artificial lipid-rich plaque, fresh bovine cardiac muscle was trimmed into a 5 × 5cm^2^ sample with a surgical blade. Mayonnaise (Kraft) as a rich source of lipid (> 80%) was injected into this sample with an insulin needle with a gauge of 30 G at a depth about 70–500 μm beneath the surface, to mimic the cap thickness of typical coronary arterial plaques^42,45^. The aorta specimen was obtained through the National disease research interchange (NDRI) from an 88-year-old female who died of cardiac disease. The study protocol was approved by York University (e2020-234 and e2020-250). A section of fresh aorta, containing palpable calcifications was prepared and presented to the imaging system on a standard glass slide and imaged immediately after preparation at room temperature.

### Imaging protocol and the datasets

PT-OCT imaging used a PT laser at 806 nm for the PDMS samples and a 1210 nm laser for the tissue samples. The laser irradiance was modulated in a sinusoidal shape from zero to a maximum power of 5 mW at 806 nm and 10mW at 1210 nm. The frequency of the modulation (*f*_*m*_) was set to either 500 or 4000 Hz. Experiments were performed with an OCT A-line rate of 21.6 kHz. In M-scanning mode, each depth profile was captured 1000 times (1000/21,600≃46ms), before moving to adjacent points to build a B-scan cross-sectional image. Each sample was imaged at various distances relative to the focal plane and with different PT power levels. Several areas were imaged for each sample.

To calculate the OCT phase signal, as illustrated in Fig. 1b, A-line signals from the sample are captured over time while the sample is exposed to the intensity-modulated PT laser (aka. M-scan). After M-scan, via standard OCT signal processing OCT amplitude and phase signals/images are calculated (Fig. 1c). Subsequently, utilizing the lock-in method (a Fourier analysis approach), the spectrum of the OCT phase, which demonstrates the amplitude of the PT-OCT signal at modulation frequency of PT laser, is computed (Fig. 1d, e). It is important to note that there was no averaging performed during the OCT phase calculation steps. The detailed signal processing can be found in Ref 10. An OCT signal intensity threshold (20 dB above noise floor) was used to mask areas of the sample without meaningful signal. These masks were applied to PT-OCT images (the green regions in Figs. 3 and 4 above the sample’s surface).

To create input signals for the neural network, time traces of the OCT phase signal were calculated. Then, the first 88 continuous datapoints were selected from the original 1000-datapoint signal to serve as input vectors to the net. Note that 88 points cover almost two modulation cycles of the photothermal modulation at a modulation frequency of 500 Hz and a sampling rate of 21.6 kHz. The amplitude of the lock-in signal from the first 864-datapoint signal was used as the GT for training the net. For comparison, lock-in analysis was done on the ST signals as well.

### The artificial neural network

The deep neural network was designed for reconstructing PT-OCT images from fast-captured signals. The structure of the network is illustrated in Fig. 2a. The network consisted of 4 layers (2 hidden layers) in a fully connected (FC) configuration. The input to the network was the 88-point time sequence of the OCT phase signal of a given pixel. The first hidden layer was a dense layer with 10 nodes. It was followed by the second dense layer with 5 nodes. The output of the network was the estimated PT-OCT amplitude. The activation function of the input layer and all hidden layers was consistently set as the rectified linear unit (ReLU) function. The activation function of the output layer was a linear function. Weight initialization throughout the entire network followed a uniformly random approach. No regularization layer, such as a dropout layer, was included in our network.

### Training of the net

In training procedure, we used k-fold cross-validation (k = 10) with mean squared error (MSE) loss function. The dataset for training was created by randomly choosing 80,000 signal traces out of 96,000 signal traces above an intensity threshold. 10 percent of the dataset (8000 signal traces) was used for validation. A PT-OCT image consisting of 100 A-lines on average has 10,000 meaningful pixels (100 × 100), so to build this library, less than 20 images were required. Training was achieved in 120 epochs with adaptive moment estimation (ADAM) optimizer. The mini-batch size was chosen as 512 for training. After completion of training of all of these 10 networks, one of them was selected randomly for hold-out testing. We used the Spyder environment powered by Python and using the Keras library. The training was performed on a GPU (GeForce GTX 1060, NVIDIA), taking only a few minutes (< 5 min) to complete. After training, to test the performance, the network was fed with unseen datasets from parts of samples other than the part used for the training. While the post-processing time for the trained network is processor-dependent, the processing time for a standard PT-OCT image (500 by 1000 pixels) was notably swift, typically less than 5 s, even when utilizing regular CPUs.

Eventually, to analyze the trained network performance, three criteria were used: Michelson contrast, MSE, and SSIM. Michelson contrast compares the contrast between two windowed images:

1 $$C_{{Michelson}} = {\raise0.7ex\hbox{${(L_{{max}} - L_{{min}} )}$} \!\mathord{\left/ {\vphantom {{(L_{{max}} - L_{{min}} )} {(L_{{max}} + L_{{min}} )}}}\right.\kern-\nulldelimiterspace} \!\lower0.7ex\hbox{${(L_{{max}} + L_{{min}} )}$}}$$

where, L_max_ and L_min_ are the maximum and minimum luminance measured across the entire selected window, respectively. For Image A and Image B, MSE is calculated as:

2 $$MSE=\left(\frac{1}{n}\right)\sum {({A}_{i}-{B}_{i})}^{2}$$

Here, *A*_*i*_ is the *i*th pixel intensity in image A,* B*_*i*_ is the corresponding value in Image B, and *n* is the number of pixels. The SSIM index between two images (A, B) of size *m* by *n*, is calculated as:

3 $$SSIM=\frac{(2{\mu }_{A}{\mu }_{B}+{C}_{1})(2{\sigma }_{AB}+{C}_{2})}{({\mu }_{A}^{2}+{\mu }_{B}^{2}+{C}_{1})({\sigma }_{A}^{2}+{\sigma }_{B}^{2}+{C}_{2})}$$

where, µA is the pixel sample mean of A, µB is the pixel sample mean of B, σ_A_^2^is the variance of A, σ_B_^2^is the variance of B, σ_AB_ is the covariance of A and B, *C*_1_ = ${({K}_{1}L)}^{2}$, *C*_2_=${({K}_{2}L)}^{2}$ are two variables to stabilize the division with weak denominator, *L* is the dynamic range of the pixel-values, and *K*_*1*_ = 0.01 and *K*_*2*_ = 0.03 by default.

## Data Availability

The datasets generated and/or analyzed during the current study are available from Nima Tabatabaei upon reasonable request.

## Code availability

The code to generate the results of this study is available from Nima Tabatabaei upon reasonable request.
